# Effects of Endovenous Radiofrequency Ablation on Right Ventricular Functions and Pulmonary Hemodynamics in Superficial Venous Insufficiency

**DOI:** 10.3390/jcdd13070294

**Published:** 2026-06-25

**Authors:** Mehmet Aslan, Mustafa Özgül, Umut Serhat Sanrı, Oğuz Karahan

**Affiliations:** 1 Department of Cardiovascular Surgery, Alanya Education and Research Hospital, Antalya 07400, Turkey; 2 Department of Cardiology, Alanya Education and Research Hospital, Antalya 07400, Turkey; 3 Department of Cardiovascular Surgery, Faculty of Medicine, Alaaddin Keykubat University, Antalya 07400, Turkey

**Keywords:** chronic venous insufficiency, endovenous radiofrequency ablation, right ventricular function, pulmonary artery pressure, systemic venous congestion, TAPSE, ventricular–arterial coupling

## Abstract

**Background**: Although chronic venous insufficiency is often treated as a localized problem, it is a systemic condition that can negatively affect cardiac hemodynamics. This study investigates the associated effects of eliminating the pathologic venous reservoir on right ventricular (RV) functions, systolic pulmonary artery pressure (*s*PAP), and inferior vena cava (IVC) diameter in patients undergoing endovenous radiofrequency ablation (RFA) for severe great saphenous vein (GSV) insufficiency. **Methods**: This retrospective observational study included 154 patients who presented between September 2023 and May 2025 with GSV insufficiency (CEAP C3-C4b) and underwent endovenous RFA. Patients with major cardiopulmonary diseases were strictly excluded. Preoperative and 6-month postoperative transthoracic echocardiography records were analyzed to evaluate RV diastolic diameter, tricuspid annular plane systolic excursion (TAPSE), *s*PAP, the TAPSE/*s*PAP ratio, and IVC diameter. **Results**: At 6 months post-RFA, compared to preoperative values, a significant decrease was detected in the mean *s*PAP (14.7 ± 2.5 vs. 11.8 ± 1.8 mmHg, *p* < 0.001) and IVC diameter (2.1 ± 0.2 vs. 1.9 ± 0.2 cm, *p* < 0.001). Furthermore, significant improvements were observed in TAPSE (20.0 ± 2.0 vs. 21.5 ± 1.8 mm, *p* < 0.001) and the TAPSE/*s*PAP ratio (1.36 ± 0.15 vs. 1.82 ± 0.18 mm/mmHg, *p* < 0.001). **Conclusions**: Endovenous RFA is associated with favorable changes in right heart parameters. Eliminating pathologic extremity blood pooling may optimize venous return kinetics and subclinically improve right ventricular–pulmonary arterial coupling.

## 1. Introduction

In traditional vascular surgery practice, chronic venous insufficiency (CVI) has historically been considered a localized disease characterized primarily by edema, hyperpigmentation, and stasis ulcers in the lower extremities. Current evidence, however, indicates that the lower extremity venous system has a tight, bidirectional relationship with right heart function, central venous pressure regulation, and pulmonary hemodynamics [1,2,3]. In patients with CVI, the dilated varicose vein bed, having lost valve function, acts as a passive “volume reservoir.” Here, massive blood pooling directly impairs effective venous return to the heart and preload dynamics [1]. Moreover, the right heart and the venous system form an uninterrupted hydrostatic column connected by delicate pressure balances. Systemic venous congestion developing secondary to right ventricular (RV) failure or pulmonary hypertension increases backward hydrostatic pressure, further worsening peripheral venous stasis and insufficiency [2,3,4]. While CVI places an additional burden on the right heart by disrupting the preload balance, an insufficient right heart creates a vicious cycle that exacerbates CVI through venous fluid overload [5,6,7].

Today, this systemic and peripheral interaction can be objectively evaluated with modern non-invasive parameters. These include right ventricular–pulmonary artery (RV–PA) coupling (e.g., TAPSE/*s*PAP ratio), which reflects the adaptation of the RV to afterload, and the venous excess ultrasound score (VExUS), which grades systemic venous congestion [8,9,10]. Furthermore, it has been demonstrated that abnormal flow patterns in the common femoral vein (CFV)—a component of the peripheral circulation—are strong indicators of increased right atrial pressure [11,12,13,14].

Although this reciprocal pathophysiological link between CVI and right heart hemodynamics has been described in the literature, a critical data gap remains in vascular surgery practice. While it is known that CVI burdens the central venous system, it has not yet been fully elucidated to what extent eliminating the massive pathologic bed in the superficial venous system via ablation can normalize the blood volume returning to the central compartment and restore impaired cardiac hemodynamics. Therefore, the aim of this study is to investigate the potential associated systemic effects of standard endovenous radiofrequency ablation (RFA) in patients with severe great saphenous vein (GSV) insufficiency. Specifically, it seeks to evaluate the impact of RFA on right ventricular function, inferior vena cava (IVC) diameter, and pulmonary hemodynamics, moving beyond the mere correction of peripheral venous hemodynamics.

## 2. Materials and Methods

### 2.1. Study Design and Population

**Figure 1:**
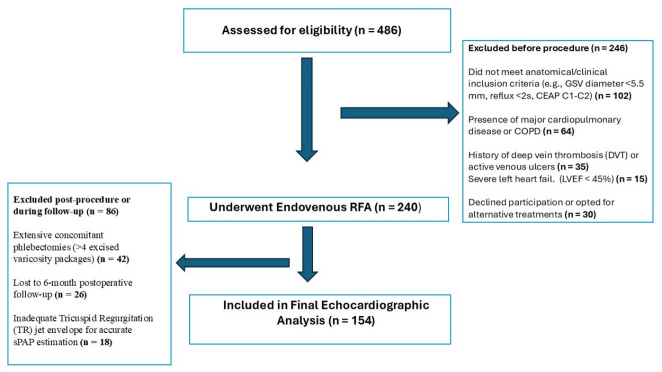
STROBE flow diagram of the study population. From an initial pool of 486 screened patients, 332 were excluded based on strict anatomical, cardiopulmonary, and methodological criteria to prevent confounding bias and ensure cohort homogeneity. The final analysis included 154 patients who underwent successful endovenous radiofrequency ablation (RFA) and completed the 6-month postoperative echocardiographic follow-up. GSV: great saphenous vein; CEAP: clinical, etiological, anatomical, and pathophysiological classification; COPD: chronic obstructive pulmonary disease; DVT: deep vein thrombosis; LVEF: left ventricular ejection fraction; RFA: radiofrequency ablation; TR: tricuspid regurgitation; *s*PAP: systolic pulmonary artery pressure.

This single-center retrospective observational clinical study evaluated the medical records of patients who presented to our clinic between September 2023 and May 2025. Initially, a total of 486 patients evaluated for symptomatic superficial venous disease were screened. After applying strict exclusion criteria to eliminate confounding cardiopulmonary factors and ensuring adequate follow-up data, a final homogenous cohort of 154 patients was included in the study (Figure 1). The study protocol was approved by the Clinical Research Ethics Committee of Alanya Alaaddin Keykubat University (Approval No.: 18-09, Date: 10 December 2025), and all procedures were conducted in accordance with the principles of the Declaration of Helsinki. Informed consent was obtained from all subjects involved in the study.

### 2.2. Inclusion and Exclusion Criteria

Inclusion criteria were as follows: (1) exhibiting a symptomatic venous disease profile classified as CEAP (clinical, etiological, anatomical, pathophysiological) stages C3, C4a, and C4b upon clinical evaluation; (2) a great saphenous vein (GSV) reflux duration of ≥2 s, indicating valve dysfunction on venous Doppler ultrasonography; and (3) a GSV diameter of ≥5.5 mm. Patients with CEAP C1 and C2 classifications were excluded because standard national health insurance reimbursement policies and surgical guidelines strictly require a GSV diameter ≥5.5 mm and a reflux duration ≥2 s for operative indication; thus, these early-stage patients are generally managed conservatively in our clinical practice. To ensure that the observed central hemodynamic variations were not confounded by pre-existing cardiopulmonary disease, we strictly excluded patients with major cardiac pathologies, severe left heart failure, idiopathic pulmonary hypertension, chronic obstructive pulmonary disease (COPD), a history of deep vein thrombosis (DVT), or active venous ulcers. To standardize variables associated with the operative technique, all patients uniformly underwent percutaneous endovenous radiofrequency ablation (RFA) under local tumescent anesthesia using the Medtronic ClosureFast™ system (Medtronic, Dublin, Ireland). Furthermore, to prevent procedural bias and ensure clinical homogeneity, patients requiring extensive concomitant phlebectomies (defined as >4 excised varicosity packages) were consciously excluded from the study. Postoperatively, all patients were prescribed Class II (20–30 mmHg) compression stockings for exactly one week—a pragmatic duration tailored to maximize patient compliance given the hot climate of our geographic region—along with standard prophylactic low-molecular-weight heparin (LMWH). Routine postoperative venous duplex ultrasound evaluations confirmed a 100% target vein occlusion rate, with no recorded incidences of deep vein thrombosis (DVT) or endovenous heat-induced thrombosis (EHIT).

### 2.3. Echocardiographic and Ultrasonographic Measurements

Standard two-dimensional transthoracic echocardiography (TTE) and color Doppler examinations were performed on all patients. Echocardiographic measurements were obtained in accordance with the American Society of Echocardiography (ASE) guidelines. Data were recorded at rest during the preparation phase (one week prior to surgery) and at the routine six-month postoperative follow-up visit. Left ventricular ejection fraction (LVEF) was measured and reported as whole numbers, reflecting standard clinical practice. To evaluate right heart function and pulmonary hemodynamics, the following specific parameters were meticulously recorded:

Right Ventricular (RV) Diastolic Diameter: Measured transversely in the basal segment from the apical four-chamber view.

Tricuspid Annular Plane Systolic Excursion (TAPSE): Measured using M-mode as the apical displacement of the lateral tricuspid annulus during systole (in mm), serving as an indicator of RV systolic function.

Systolic Pulmonary Artery Pressure (*s*PAP): Calculated via the modified Bernoulli equation using the maximum tricuspid regurgitation velocity measured by continuous-wave Doppler, incorporating the estimated right atrial pressure (RAP) derived from IVC diameter and collapsibility characteristics.

Inferior Vena Cava (IVC) Diameter: Measured in centimeters from the subcostal view, just proximal to its junction with the right atrium, to estimate right atrial pressure.

TAPSE/*s*PAP Ratio: Calculated as the ratio of TAPSE to *s*PAP, serving as a non-invasive surrogate marker for right ventricular–pulmonary arterial (RV-PA) coupling.

### 2.4. Statistical Analysis

Statistical analyses and data visualization were performed using SPSS software, version 29.0 (IBM Corp., Armonk, NY, USA), and GraphPad Prism, version 8.4 (GraphPad Software, San Diego, CA, USA). Continuous variables were assessed for normal distribution and are presented as mean ± standard deviation (SD). Categorical variables are expressed as frequencies (*n*) and percentages (%). A paired-sample *t*-test was used to compare the preoperative and six-month postoperative continuous echocardiographic measurements. A *p*-value of <0.05 was considered statistically significant.

## 3. Results

A total of 154 patients who met the inclusion criteria and completed the six-month follow-up were included in the final analysis. The cohort consisted of 88 (57.1%) males and 66 (42.9%) females, with a mean age of 53.6 ± 11.2 years. The mean body mass index (BMI) was 26.8 ± 3.5 kg/m^2^. According to the CEAP classification, 52 (33.8%) patients had a C3, 48 (31.2%) had a C4a, and 54 (35.0%) had a C4b disease profile. Regarding comorbidities, 22 (14.3%) patients had hypertension (HT), 12 (7.8%) had diabetes mellitus (DM), and 15 (9.7%) had hyperlipidemia. Preoperative routine TTE evaluations revealed preserved left ventricular systolic function across the majority of the cohort, with a mean left ventricular ejection fraction (LVEF) of 55.7 ± 5.1%. Although 4 elderly patients exhibited a baseline LVEF between 45% and 49%, they were closely monitored and initially included to avoid selection bias among patients with complete follow-ups. A post hoc sensitivity analysis excluding these 4 patients demonstrated that the statistical significance of the primary right heart outcomes remained robustly unchanged (all *p* < 0.001). Upon ultrasonographic vascular mapping, the mean diameter of the targeted pathologic GSV segment was 9.0 ± 2.0 mm, and the mean valve reflux duration was 3.8 ± 1.0 s (Table 1).

### Postoperative Right Cardiac and Pulmonary Hemodynamic Changes

In all patients, the pathologic reservoir bed causing severe superficial venous insufficiency (GSV) was successfully ablated using endovenous RFA. A comparison of the preoperative and six-month postoperative echocardiographic data revealed a highly significant decrease in pulmonary artery pressure. Specifically, the mean baseline *s*PAP significantly regressed from 14.7 ± 2.5 mmHg to 11.8 ± 1.8 mmHg at six months post-intervention (*p* < 0.001).

**Figure 2:**
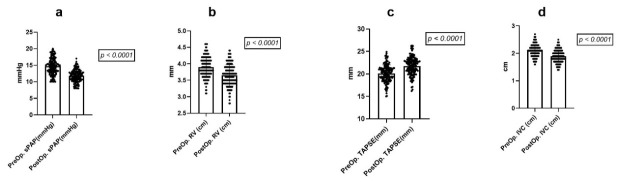
Hemodynamic and cardiac parameter changes following endovenous RFA. Individual pre- and postoperative changes in (**a**) systolic pulmonary artery pressure (*s*PAP); (**b**) right ventricular (RV) diastolic diameter; (**c**) tricuspid annular plane systolic excursion (TAPSE); and (**d**) inferior vena cava (IVC) diameter. Dots represent individual data points, horizontal lines indicate the mean, and error bars represent the standard deviation (SD). Exact *p*-values indicating statistical significance are displayed above the respective plots.

This significant reduction in pulmonary pressure was accompanied by improvements in other key right heart and central venous parameters. A statistically significant reduction was observed in the IVC diameter—a direct indicator of central venous congestion (from 2.1 ± 0.2 to 1.9 ± 0.2 cm, *p* < 0.001). Furthermore, TAPSE, which reflects the systolic contractile strength of the right ventricle, significantly increased (from 20.0 ± 2.0 to 21.5 ± 1.8 mm, *p* < 0.001). Additionally, the RV diastolic diameter significantly decreased (from 3.9 ± 0.3 to 3.7 ± 0.3 cm, *p* < 0.001) (Figure 2). Consequently, right ventricular–pulmonary arterial (RV-PA) coupling demonstrated significant improvement, as evidenced by the increase in the mean TAPSE/*s*PAP ratio from 1.36 ± 0.15 to 1.82 ± 0.18 mm/mmHg at the six-month follow-up (*p* < 0.001) [Table 2].

## 4. Discussion

This study reveals that endovenous radiofrequency ablation (RFA), conventionally applied in vascular surgery practice solely to correct local peripheral venous hemodynamics, also exerts significant associated decongestive effects on right heart function and central venous dynamics. Following the RFA procedure in patients with advanced GSV insufficiency, a narrowing of the inferior vena cava (IVC) diameter, a significant decrease in mean systolic pulmonary artery pressure (*s*PAP) (from 14.7 to 11.8 mmHg), and an increase in the TAPSE value (to 21.5 mm)—reflecting enhanced right ventricular systolic function—were detected. These findings demonstrate that restoring the peripheral venous bed to its physiological limits directly alleviates central congestion and the subclinical workload on the right side of the heart.

Physiologically, in advanced-stage venous insufficiency, dilated varicose veins act as a massive venous “capacitance reservoir” under high hydrostatic pressure [15]. This pooled volume impairs effective venous return to the heart, reduces IVC collapsibility, and negatively affects early diastolic filling. In our study, the regression of the IVC diameter to physiological limits (mean 1.9 cm) in the postoperative period suggests that ablating this pathologic venous pool provides a systemic “decongestion” effect and optimizes preload. This is pathophysiologically consistent with current data in the intensive care literature, which show that fluid challenge worsens venous congestion and portal pulsatility [16,17]. Indeed, as demonstrated in the landmark studies by Mullens et al., the main culprit of target organ function deterioration in advanced heart failure is not decreased cardiac output (forward failure) but rather directly increased central venous pressure and systemic venous congestion [18].

By thermally ablating the incompetent superficial segments, the total circulating blood volume is not reduced; rather, the pathological hydrostatic column is eliminated, and the efficiency of the calf-muscle pump is restored. This prevents excessive and dyssynchronous volume fluctuations from reaching the right atrium. In our study, the concurrent decrease in *s*PAP and increase in TAPSE indicate that right ventricular–pulmonary arterial (RV–PA) coupling was restored at the clinical level. This is a critical parameter reflecting the energy transfer from the right ventricle to the pulmonary vascular bed, validated in clinical practice by the improved TAPSE/*s*PAP ratio [9].

Although the reduction in mean *s*PAP (from 14.7 to 11.8 mmHg) is statistically significant, both values remain within the normal physiological range. While a ~3 mmHg reduction does not constitute a clinical cure for pulmonary hypertension, it represents a subclinical shift toward optimized RV-PA coupling. Early intervention may thus prevent long-term maladaptive remodeling before overt right heart strain develops, though intraobserver variability in echocardiographic *s*PAP estimations must be acknowledged. Although the right ventricle initially exhibits adaptive remodeling in response to volume or pressure overload, the chronicity of this burden eventually evolves into maladaptive processes and cardiac fatigue [19]. As a highly pressure- and volume-sensitive pump, the right ventricle can exhibit dyssynchronous contractions when exposed to increased or fluctuating venous return [20,21,22]. When the insufficient GSV segment is thermally ablated with RFA, the continuous and turbulent hydrostatic column reflecting from the periphery to the center is eliminated. Consequently, while secondary pressure on the pulmonary vascular bed is alleviated, the systolic excursion capacity of the RV increases [4,19].

In light of these systemic mechanisms, the present findings necessitate a paradigm shift in the clinical management of CVI. Current evidence reveals a bidirectional, reciprocal interaction between heart failure and chronic venous insufficiency that disrupts the venous return and loading dynamics of the heart [1]. Right ventricular dysfunction developing secondary to left or right heart diseases increases backward hydrostatic pressure, further worsening peripheral venous stasis [3]. It has been shown that the frequency of lower extremity venous insufficiency insidiously increases even in primary pulmonary arterial hypertension patients with fully preserved right ventricular function [2]. Delaying the surgical treatment of patients with advanced venous insufficiency on the grounds that they are asymptomatic may pave the way not only for the progression of regional findings but also for the deterioration of right ventricular mechanics over time, leading to a progressive rise in pulmonary artery pressure. Recent studies have confirmed that abnormal Doppler flow patterns in the common femoral vein (CFV) are strong indicators of both increased right atrial pressure [15] and severe pulmonary hypertension [23]. In future clinical practice, evaluating CVI patients for multisystemic venous parameters via preoperative echocardiography might provide valuable associative insights, highlighting the potential systemic benefits of superficial venous ablation.

### Study Limitations

The present study has several limitations. First, its single-center retrospective design, lack of echocardiographic blinding, and lack of a control group (e.g., conservatively managed CVI patients with compression therapy alone) limit strict causal inference. Multicenter prospective studies are needed to confirm the generalizability of these findings. Second, potential confounding variables—such as the use of diuretics, antihypertensive therapy, and postoperative weight changes—were not strictly controlled during the follow-up period. Third, because our cohort was carefully selected from an initial pool of 486 patients to exclude major cardiopulmonary diseases, the final 154 patients had baseline cardiac parameters well within normal limits. Due to this extreme homogeneity and the absence of clinical failure, there was insufficient statistical variance to robustly identify independently distinct subgroups via multivariable regression. Additionally, right heart function and pulmonary artery pressure (*s*PAP) were evaluated using non-invasive echocardiographic measurements rather than invasive right heart catheterization, which remains the gold standard. Standard IVC diameter measurements were used as an isolated parameter to evaluate central congestion; however, current literature predicts that the accuracy of these measurements will become increasingly standardized with emerging automated edge-tracking echocardiography algorithms [24]. Although comprehensive multi-organ congestion scoring systems, such as VExUS, have been described, they are not utilized in routine vascular surgery outpatient practice and fall outside the pragmatic clinical scope of this study. Moreover, the absence of standard venous clinical endpoints (e.g., Venous Clinical Severity Score [VCSS]) is an additional limitation, as our study focused strictly on objective hemodynamic parameters. Finally, longer-term follow-up data are required to confirm the permanence of the structural reverse remodeling achieved in the right ventricle.

## 5. Conclusions

Chronic venous insufficiency is not a localized anatomical disorder limited to the lower extremities; it is a dynamic circulatory disease that directly impairs cardiopulmonary hemodynamics by triggering systemic venous congestion. Endovenous RFA applied in patients with advanced GSV insufficiency successfully regulates abnormal venous loading dynamics on the heart by eliminating the pathologic volume pool in the lower extremities. The observed postoperative narrowing of the IVC diameter, the decrease in *s*PAP, and the significant improvements in both TAPSE and the TAPSE/*s*PAP ratio suggest that venous ablation is associated with improved ventricular–arterial coupling at a subclinical level. In this context, we suggest that endovenous ablation in vascular surgery practice should be adopted not merely as a peripheral procedure to relieve local symptoms but as a proactive intervention that provides favorable systemic cardiopulmonary hemodynamic changes.

## Figures and Tables

**Table 1:** Demographic and clinical characteristics of the patients. The table presents baseline demographic data (age, gender, and BMI), CEAP clinical classification, comorbidities, and preoperative basal echocardiographic/ultrasonographic measurements of the 154 patients. Categorical variables are expressed as numbers (percentages) and continuous variables as mean ± standard deviation.

Parameter	Value (*n* = 154)
Age (Years)	53.6 ± 11.2
Gender (Male/Female)	88 (57.1%)/66 (42.9%)
Body Mass Index (kg/m^2^)	26.8 ± 3.5
CEAP Stage (C3/C4a/C4b)	52 (33.8%)/48 (31.2%)/54 (35.0%)
Comorbidities	
-Hypertension (HT)	22 (14.3%)
-Diabetes Mellitus (DM)	12 (7.8%)
-Hyperlipidemia	15 (9.7%)
Left Ventricular EF (%)	55.7 ± 5.1 (Min: 45, Max: 68)
Preoperative GSV Diameter (mm)	9.0 ± 2.0
Preoperative Reflux Duration (sec)	3.8 ± 1.0
Operation Type (RFA)	154 (100%)

**Table 2:** Comparison of preoperative and postoperative echocardiographic parameters. Comparative analysis of parameters indicating right heart functions (TAPSE, RV diameter) and pulmonary hemodynamics (*s*PAP, IVC diameter) before endovenous RFA and at the 6th postoperative month. A *p*-value of <0.05 was considered statistically significant.

Parameter	Preoperative	Postoperative (6th Month)	*p*-Value
Systolic Pulmonary Artery Pressure (*s*PAP, mmHg)	14.7 ± 2.5	11.8 ± 1.8	<0.001
Right Ventricular Diastolic Diameter (cm)	3.9 ± 0.3	3.7 ± 0.3	<0.001
TAPSE (mm)	20.0 ± 2.0	21.5 ± 1.8	<0.001
Inferior Vena Cava (IVC) Diameter (cm)	2.1 ± 0.2	1.9 ± 0.2	<0.001
TAPSE/*s*PAP Ratio (mm/mmHg)	1.36 ± 0.15	1.82 ± 0.18	<0.001

## Data Availability

The data presented in this study are available upon request from the corresponding author.

## References

[1] Damay V.A., Ivan I., Benitez V.M.C. (2025). Chronic venous insufficiency in heart failure: Exploring a reciprocal influence on cardiovascular health. Heart Fail. Rev..

[2] Aldemir M., Emren S.V., Balçık Ç., Onrat E., Gürsoy M. (2018). Primary pulmonary arterial hypertension with preserved right ventricular function leads to lower extremity venous insufficiency. Vascular.

[3] Dini F.L., Pugliese N.R., Ameri P., Attanasio U., Badagliacca R., Correale M., Mercurio V., Tocchetti C.G., Agostoni P., Palazzuoli A. (2023). Right ventricular failure in left heart disease: From pathophysiology to clinical manifestations and prognosis. Heart Fail. Rev..

[4] Konstam M.A., Kiernan M.S., Bernstein D., Bozkurt B., Jacob M., Kapur N.K., Kociol R.D., Lewis E.F., Mehra M.R., Pagani F.D. (2018). Evaluation and Management of Right-Sided Heart Failure: A Scientific Statement from the American Heart Association. Circulation.

[5] Huard K., Lachance O., Parent M., Tawil P., Saade E., Hammoud A., Couture E.J., Lamarche Y., Jarry S., Calderone A. (2025). Prevalence of abnormal common femoral vein pulsatility on Doppler ultrasound in patients undergoing cardiac surgery and its association with adverse events: A prospective cohort study. Can. J. Anaesth..

[6] Dias N.H., Gomes D.R., de Oliveira A.C.T., Pellegrini J.A.S., Boniatti M.M. (2023). Prognostic value of Doppler waveform analysis of common femoral vein in septic patients: A prospective cohort study. J. Ultrasound.

[7] Mazzolai L., Aboyans V., Ageno W., Agnelli G., Alatri A., Bauersachs R., Brekelmans M.P.A., Büller H.R., Elias A., Farge D. (2018). Diagnosis and management of acute deep vein thrombosis: A joint consensus document from the European Society of Cardiology working groups of aorta and peripheral vascular diseases and pulmonary circulation and right ventricular function. Eur. Heart J..

[8] Koratala A., Romero-González G., Soliman-Aboumarie H., Kazory A. (2024). Unlocking the Potential of VExUS in Assessing Venous Congestion: The Art of Doing It Right. Cardiorenal Med..

[9] Tello K., Wan J., Dalmer A., Vanderpool R., Ghofrani H.A., Naeije R., Roller F., Mohajerani E., Seeger W., Herberg U. (2019). Validation of the Tricuspid Annular Plane Systolic Excursion/Systolic Pulmonary Artery Pressure Ratio for the Assessment of Right Ventricular-Arterial Coupling in Severe Pulmonary Hypertension. Circ. Cardiovasc. Imaging.

[10] Eljaiek R., Cavayas Y.A., Rodrigue E., Desjardins G., Lamarche Y., Toupin F., Denault A.Y., Beaubien-Souligny W. (2019). High postoperative portal venous flow pulsatility indicates right ventricular dysfunction and predicts complications in cardiac surgery patients. Br. J. Anaesth..

[11] Croquette M., Larrieu Ardilouze E., Beaufort C., Jutant E.M., Puyade M., Montani D., Thollot C., Lanéelle D., De Géa M., Trihan J.E. (2025). Femoral venous stasis index predicts elevated right atrial pressure and mortality in pulmonary hypertension. ERJ Open Res..

[12] Croquette M., Puyade M., Montani D., Jutant E.M., De Géa M., Lanéelle D., Thollot C., Trihan J.E. (2023). Diagnostic Performance of Pulsed Doppler Ultrasound of the Common Femoral Vein to Detect Elevated Right Atrial Pressure in Pulmonary Hypertension. J. Cardiovasc. Transl. Res..

[13] Bhardwaj V., Rola P., Denault A., Vikneswaran G., Spiegel R. (2023). Femoral vein pulsatility: A simple tool for venous congestion assessment. Ultrasound J..

[14] Bhardwaj V., Samprathi A., Saha K., Orozco N., Hegde P., Nizamudin M., Chacko J., Varma M.M.K., Denault A., G V. (2025). Dual doppler dynamics: Integrating femoral venous doppler and VExUS for predicting organ dysfunction in acute heart failure. J. Anesth. Analg. Crit. Care.

[15] Melo R.H., Wong A., Koratala A., Kattan E., da Hora Passos R. (2026). Femoral vein Doppler ultrasound for assessing venous congestion and right heart function: A scoping review. Intensive Care Med. Exp..

[16] Koratala A. (2026). Extended venous excess ultrasound: A promising addition to point-of-care ultrasound-based venous congestion assessment. World J. Cardiol..

[17] Bitar Z.I., Maadarani O.S., Bitar M. (2025). Ultrasound indicators of organ venous congestion: A narrative review. Ann. Intensive Care.

[18] Mullens W., Abrahams Z., Francis G.S., Sokos G., Taylor D.O., Starling R.C., Young J.B., Tang W.H.W. (2009). Importance of venous congestion for worsening of renal function in advanced decompensated heart failure. J. Am. Coll. Cardiol..

[19] Rako Z.A., Kremer N., Yogeswaran A., Richter M.J., Tello K. (2023). Adaptive versus maladaptive right ventricular remodelling. ESC Heart Fail..

[20] Ewalts M., Dawkins T., Boulet L.M., Thijssen D., Stembridge M. (2021). The influence of increased venous return on right ventricular dyssynchrony during acute and sustained hypoxaemia. Exp. Physiol..

[21] Giangregorio F., Centenara E., Mazzocchi S., Gerra L., Tursi F., Imberti D., Aschieri D. (2025). Assessing Venous Congestion in Acute and Chronic Heart Failure: A Review of Splanchnic, Cardiac and Pulmonary Ultrasound: Part 1: Conventional B-Mode, Colordoppler, and Vexus Protocol. J. Clin. Med..

[22] Giangregorio F., Centenara E., Mazzocchi S., Gerra L., Tursi F., Imberti D., Aschieri D. (2026). Assessing Venous Congestion in Heart Failure: A Review of Splanchnic, Cardiac, and Pulmonary Ultrasound: Part 2: Contrast-Enhanced Ultrasound and Shear Wave. J. Clin. Med..

[23] Torres-Arrese M., Barberá-Rausell P., Li-Zhu J.O., Salas-Dueñas R., Real-Martín A.E., Mata-Martínez A., Gonzalo-Moreno B., Núñez J.H., Luordo D., Cano J.G.S. (2024). The Cardiac Pulsed Wave Doppler Pattern of the Common Femoral Vein in Diagnosing the Likelihood of Severe Pulmonary Hypertension: Results from a Prospective Multicentric Study. J. Clin. Med..

[24] Albani S., Mesin L., Roatta S., De Luca A., Giannoni A., Stolfo D., Biava L., Bonino C., Contu L., Pelloni E. (2022). Inferior Vena Cava Edge Tracking Echocardiography: A Promising Tool with Applications in Multiple Clinical Settings. Diagnostics.

